# A clinical informatics approach to bronchopulmonary dysplasia: current barriers and future possibilities

**DOI:** 10.3389/fped.2024.1221863

**Published:** 2024-02-12

**Authors:** Alvaro G. Moreira, Ameena Husain, Lindsey A. Knake, Khyzer Aziz, Kelsey Simek, Charles T. Valadie, Nisha Reddy Pandillapalli, Vanessa Trivino, James S Barry

**Affiliations:** ^1^ Department of Pediatrics, University of Texas Health San Antonio, San Antonio, TX, United States; ^2^ Department of Pediatrics, University of Utah, Salt Lake City, UT, United States; ^3^ Department of Pediatrics, University of Iowa, Iowa City, IA, United States; ^4^ Department of Pediatrics, Johns Hopkins University, Baltimore, MD, United States; ^5^ Department of Pediatrics, University of Colorado School of Medicine, Aurora, CO, United States

**Keywords:** informatics, bronchopulmonary dysplasia, chronic lung disease, premature neonate, clinical decision

## Abstract

Bronchopulmonary dysplasia (BPD) is a complex, multifactorial lung disease affecting preterm neonates that can result in long-term pulmonary and non-pulmonary complications. Current therapies mainly focus on symptom management after the development of BPD, indicating a need for innovative approaches to predict and identify neonates who would benefit most from targeted or earlier interventions. Clinical informatics, a subfield of biomedical informatics, is transforming healthcare by integrating computational methods with patient data to improve patient outcomes. The application of clinical informatics to develop and enhance clinical therapies for BPD presents opportunities by leveraging electronic health record data, applying machine learning algorithms, and implementing clinical decision support systems. This review highlights the current barriers and the future potential of clinical informatics in identifying clinically relevant BPD phenotypes and developing clinical decision support tools to improve the management of extremely preterm neonates developing or with established BPD. However, the full potential of clinical informatics in advancing our understanding of BPD with the goal of improving patient outcomes cannot be achieved unless we address current challenges such as data collection, storage, privacy, and inherent data bias.

## 1 Incidence/prevalence of BPD and why it is a priority for improvement in neonatal care and outcomes

Bronchopulmonary dysplasia (BPD) is a chronic lung disease affecting extremely preterm neonates, characterized by disruptions in the development of the alveolar and vascular compartments leading to long-term pulmonary complications (1–3). Despite significant progress in neonatal care, the incidence of BPD has remained relatively unchanged, particularly with the increasing survival rates of extremely preterm neonates. To put this problem into perspective, it is essential to note that BPD represents a substantial burden on both healthcare systems (e.g., up to $442,468 per neonate in the first year of life) and affected families (4). The financial healthcare costs associated with managing BPD, coupled with the emotional and physical toll it takes on families, underscore the urgency of addressing this condition.

Interestingly, some studies have suggested that the incidence of BPD may be decreasing among moderately preterm infants, possibly due to improved respiratory support techniques, antenatal steroids, and surfactant replacement therapy (5). However, this positive trend might not hold true for extremely preterm infants. This evolving scenario underscores the importance of innovative approaches as current therapies (e.g., oxygen, bronchodilators, and glucocorticoids) have limited benefits apart from symptom management. There is a critical need not only for newer, more effective therapies for BPD but also for approaches that may predict and potentially identify neonates who would benefit most from targeted or earlier interventions (6–8). By diligently tracking these trends and facilitating personalized care strategies for infants at risk of BPD, we can better navigate the complex dynamics of BPD incidence and improve both the prevention and management of this challenging condition.

**Figure 1:**
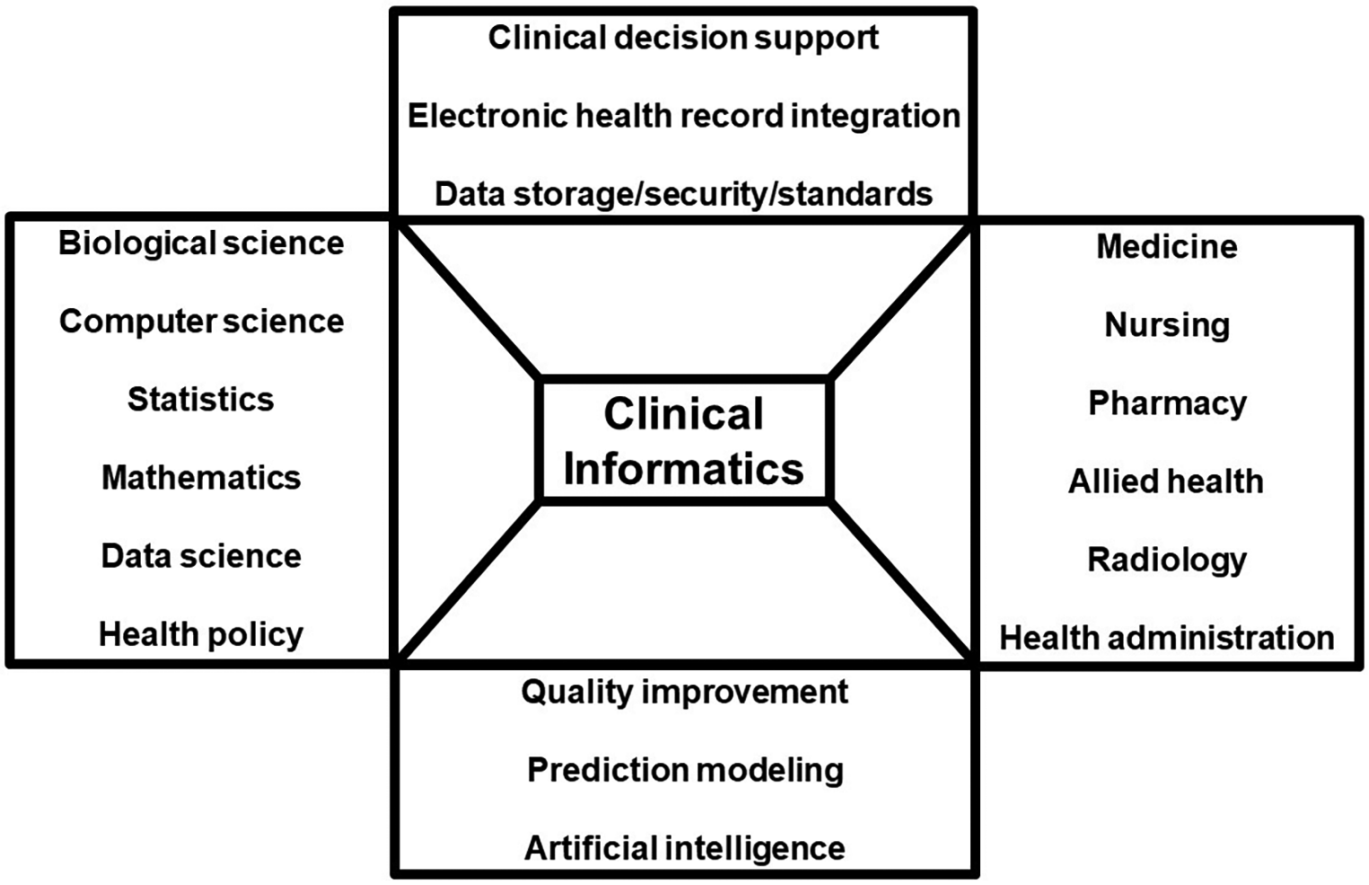
Schematic demonstrating disciplines and applications of clinical informatics.

Clinical informatics, a rapidly expanding field in biomedical informatics, is transforming healthcare by integrating computational methods with patient data to improve outcomes [(9–11), Figure 1]. This approach has demonstrated significant success in various areas of healthcare. For instance, clinical informatics has played a pivotal role in advancing cancer care, where it facilitates precision medicine by tailoring treatments based on genetic and clinical data, ultimately leading to more effective therapies and improved patient outcomes (12–14). In the realm of critical care medicine, clinical informatics has led to enhanced patient monitoring systems, predictive analytics for the early detection of deteriorating conditions, and streamlined communication among healthcare teams, resulting in better patient management and reduced mortality rates (15–17).

Within neonatal care, clinical informatics has made substantial contributions as well. Two notable examples include retinopathy of prematurity (ROP) screening and neonatal intensive care unit (NICU) workflow optimization. Clinical informatics has been instrumental in developing algorithms that analyze retinal images to identify preterm infants at risk of ROP. By automating this process, clinicians can make quicker and more accurate decisions about when to initiate treatment, preventing vision loss in vulnerable neonates (18–20). Clinical informatics tools have also been employed to optimize workflows in the NICU. These tools assist in managing patient data, monitoring vital signs, and coordinating care among healthcare professionals. Such optimization not only improves the quality of care but also reduces the burden on healthcare providers (21–24). These examples illustrate how clinical informatics is revolutionizing healthcare by harnessing data-driven approaches to enhance patient outcomes across a wide range of medical specialties.

In terms of BPD, clinical informatics presents new and exciting opportunities to enhance clinical therapy. For example, leveraging electronic health records (EHRs) or continuous vital sign data to capture nuanced ventilator or physiologic data can aid in identifying patients for targeted approaches. Machine learning algorithms can also analyze EHRs to identify complex patterns that may predict or subclassify BPD or provide prognostic measures for long-term respiratory outcomes [e.g., need for home oxygen, tracheostomy (25–28)]. Beyond EHRs, mobile health and clinical decision support systems are additional clinical informatics tools that can benefit neonates with BPD.

This review highlights the innovative potential of clinical informatics in improving the management of BPD, specifically in predicting extubation success, identifying clinically relevant phenotypes, and developing clinical decision support tools. While these informatics tools offer a promising avenue to provide clinicians with more accurate and timely information, several challenges need to be addressed. It is important to note that one significant hurdle is the collection of data from multiple institutions. Collaborative efforts to aggregate data from various healthcare facilities can enhance the generalizability of clinical and research findings, enabling a broader understanding of BPD. Additionally, the need for ethical and secure storage of data generated by EHRs poses a critical challenge. Ensuring patient privacy and data security is paramount in clinical informatics, especially when dealing with sensitive neonatal information. By proactively addressing these challenges, clinical informatics can play a pivotal role in advancing our understanding of BPD in neonates and improving patient outcomes. The collaboration among institutions and the implementation of robust data security measures are crucial steps toward harnessing the full potential of informatics tools in enhancing neonatal care for BPD.

## 2 The lack of advances in the therapeutic management of BPD or its prevention

The limited advances in the treatment and prevention of BPD can be attributed to several interconnected factors within the field of neonatal care. Firstly, the multifactorial nature of BPD, which involves genetic predisposition, developmental lung immaturity, and various environmental factors, makes it a complex and challenging condition to address comprehensively (29–31). These intricate interactions have hindered the development of targeted therapies, as the underlying mechanisms of BPD remain incompletely understood.

Secondly, the lack of effective interventions to modify the natural course of BPD in its early stages has contributed to the stagnation in therapeutic progress (32). Current treatment options primarily focus on managing symptoms and providing respiratory support, but there is a significant gap in therapies that can halt or reverse the disease progression. This limitation is compounded by the vulnerability of extremely preterm neonates, who often face multiple health challenges beyond BPD, making it challenging to prioritize research efforts in this specific area.

Moreover, conducting clinical trials and research involving neonates, especially extremely preterm infants, presents ethical and logistical challenges (33). The need to balance the potential benefits of experimental treatments with the vulnerability of this patient population has led to cautious and conservative approaches in clinical research. As a result, the development and testing of novel therapeutic strategies for BPD have been slow-paced.

The financial burden associated with neonatal care, particularly the management of BPD, has also played a role in the limited advances in this field (34). Healthcare systems are often strained by the high costs of caring for extremely preterm infants, diverting resources away from research and innovation. This financial constraint has hindered the pursuit of new therapeutic avenues for BPD.

In summary, the relatively slow progress in treating and preventing BPD can be attributed to the complexity of the condition, the lack of effective interventions, ethical considerations in neonatal research, and financial constraints on healthcare systems. Addressing these challenges will be crucial in advancing our understanding of BPD and developing more effective therapies for affected neonates.

## 3 The role of clinical informatics in other healthcare fields

Clinical informatics plays a pivotal role in healthcare by integrating advanced computational methods with patient data to improve patient outcomes and enhance the overall quality of care. This rapidly evolving field harnesses the power of data analysis, EHRs, machine learning, and artificial intelligence (AI) to transform healthcare delivery across various medical specialties. This paradigm shift toward data-driven decision-making has proven particularly effective in oncology, where precision medicine and personalized treatment plans are revolutionizing cancer care.

In cancer treatment, clinical informatics has emerged as a game-changer. By analyzing vast datasets encompassing genetic, genomic, and clinical information, healthcare providers can tailor treatments to the specific genetic and molecular characteristics of a patient's cancer. This approach, known as precision oncology, has led to the development of targeted therapies that are more effective and less toxic than traditional chemotherapy. For example, drugs like Herceptin (trastuzumab) have been successful in treating breast cancer patients with HER2-positive tumors (35).

Furthermore, clinical informatics aids in the identification of novel therapeutic targets and the prediction of treatment responses. Through the analysis of genomic data, researchers can pinpoint genetic mutations that drive cancer growth and progression. This knowledge enables the development of new drugs and treatment strategies that directly target these mutations, such as the use of tyrosine kinase inhibitors in the treatment of chronic myeloid leukemia (CML) (36).

Additionally, clinical informatics facilitates the management of large patient data within oncology departments and research institutions. EHRs help streamline the recording and retrieval of patient information, ensuring that healthcare providers have access to up-to-date and comprehensive data and protocols to make informed decisions. This not only improves patient care but also aids in research by providing a wealth of structured data for retrospective analyses and future clinical trials (37). In summary, clinical informatics is reshaping the landscape of healthcare, with cancer treatment serving as a prime example of its transformative potential. By leveraging data-driven insights, precision medicine, and streamlined data management, clinical informatics is improving patient outcomes and transforming the way healthcare is delivered.

## 4 Some examples of clinical informatics used in neonatology

Neonatal nutrition is a critical aspect of the care of premature infants, who often require specialized feeding plans. Clinical informatics has helped optimize the nutritional support provided to these vulnerable neonates. EHRs enable healthcare providers to monitor and track the nutritional intake of neonates accurately. These systems allow for the precise recording of feeding volumes, nutritional supplements, growth curves, and caloric intake. By analyzing this data, healthcare teams can tailor nutrition plans to meet the specific needs of each neonate, ensuring they receive adequate nourishment to support growth and development (38). Through data analysis, informatics tools can identify trends in neonatal growth and nutritional status. If a neonate's growth trajectory deviates from the expected, the system can generate alerts, prompting healthcare providers to assess and adjust the nutrition plan accordingly. This proactive approach helps prevent undernutrition and its associated complications in neonates (39, 40).

Another practical example of clinical informatics in neonatology involves neonatal infection control, a significant concern when caring for premature infants. EHRs play a central role in tracking and monitoring neonatal infections. These systems allow healthcare providers to record and analyze data related to infection risk factors, microbiology results, antibiotic usage, and infection outcomes (41). Informatics tools can automatically generate alerts when certain infection risk factors are identified, such as prolonged antibiotic use or central line-associated bloodstream infections (CLABSIs). These alerts prompt healthcare teams to take real-time action, such as adjusting antibiotic regimens or implementing infection control measures. Additionally, informatics systems facilitate the timely retrieval of microbiology results, aiding clinicians in diagnosing and treating infections promptly (42).

Furthermore, clinical informatics supports the surveillance and reporting of neonatal infection rates. By aggregating and analyzing infection data from multiple NICUs, informatics tools enable healthcare professionals to identify trends, outbreaks, and areas for improvement in infection control practices. This data-driven approach helps NICUs implement evidence-based strategies to reduce infection rates and improve neonatal outcomes (43). The Kaiser Permanente sepsis calculator, which has now been implemented in some EHRs, has also helped clinicians calculate risk (44).

## 5 Exploring the role of clinical informatics in the prevention and treatment of BPD is a logical and forward-thinking endeavor

Clinical informatics has demonstrated its transformative potential in various healthcare domains, making it prudent to explore its role in BPD prevention and treatment.

### 5.1 The data-driven imperative

Clinical informatics is fundamentally data-driven, harnessing the power of EHRs, computational methods, and artificial intelligence to derive meaningful insights from a vast array of data. In the case of BPD, the substantial amount of data available in neonatal care can be leveraged to gain a deeper understanding of the condition. This method can elucidate hidden patterns, identify risk factors, and inform evidence-based interventions, potentially modernizing our approach to BPD (45).

### 5.2 Personalized care for neonates

BPD is not a uniform condition, and neonates exhibit considerable variability in their response to treatments and outcomes. Clinical informatics offers the promise of personalized medicine tailored to the unique needs of each neonate. By analyzing patient-specific data, including genetic markers, clinical history, and physiological parameters, informatics tools can help clinicians predict which neonates are at the highest risk of developing severe BPD and tailor interventions accordingly. This personalized approach aligns with the growing trend in medicine toward precision care and could lead to more effective treatments for BPD (46, 47).

### 5.3 Tracking trends and outcomes

Clinical informatics enables the systematic monitoring of trends and outcomes over time. This capability is invaluable in the context of BPD, where understanding the evolving landscape of the condition is critical. By analyzing large-scale data from multiple neonatal care units, informatics tools can help researchers and clinicians identify shifts in BPD incidence, potentially pinpoint areas with rising prevalence, and assess the effectiveness of various interventions. Such insights can guide healthcare policies and resource allocation, ultimately improving BPD prevention and management strategies (48–50).

### 5.4 Clinical decision support

In neonatal care, where rapid interventions can be life-saving, informatics tools can provide clinicians with timely information and decision-support algorithms. For example, predictive models integrated into EHRs can help identify neonates at a high risk of developing severe BPD, prompting clinicians to implement preventive measures or consider alternative treatment strategies. This proactive approach can potentially reduce the severity of BPD, improve long-term outcomes for affected neonates, and reduce healthcare costs (51, 52).

**Figure 2:**
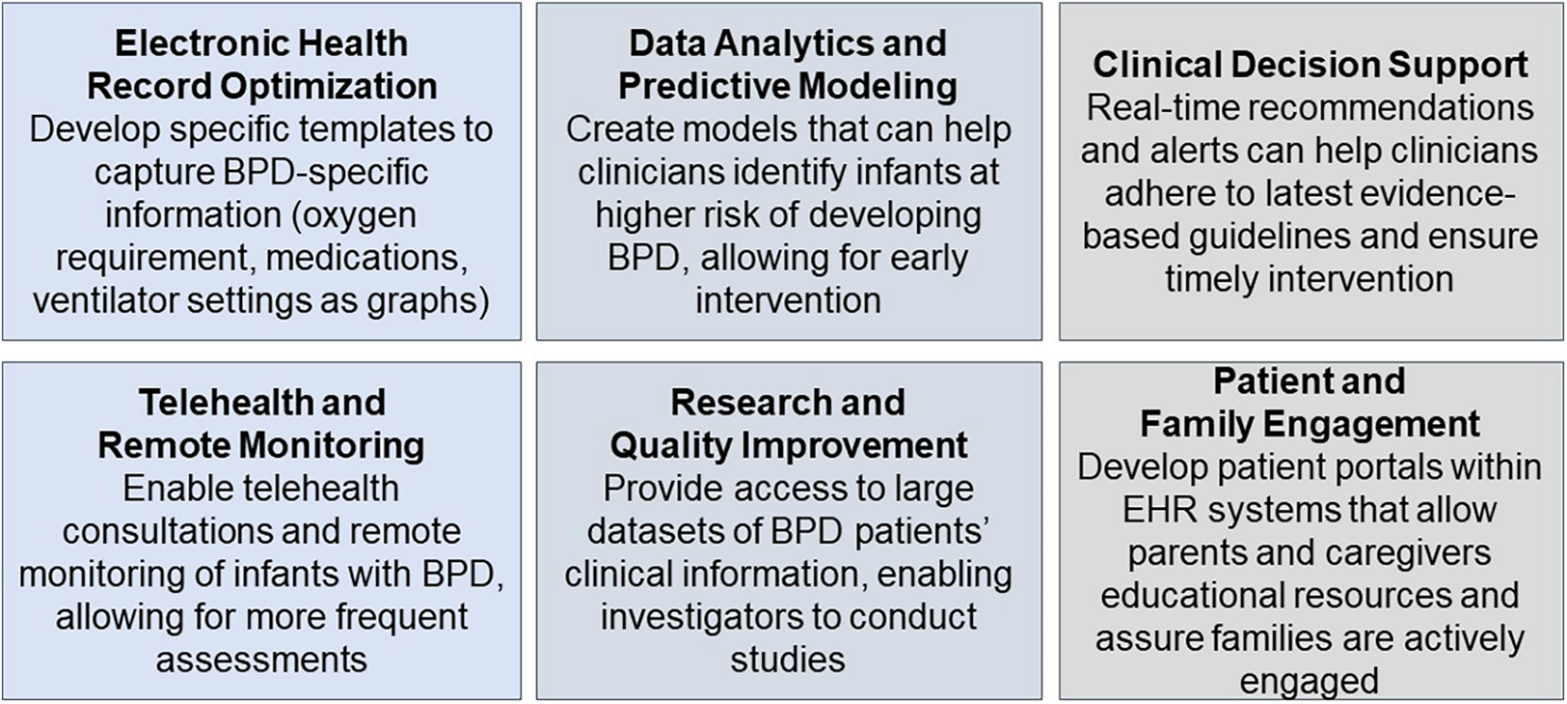
Application of clinical informatics for BPD.

In conclusion, exploring the role of clinical informatics in BPD prevention and treatment is a promising avenue that aligns with the evolving landscape of healthcare. Leveraging data-driven insights, personalizing care, tracking trends, and providing clinical decision support can improve how we approach BPD (Figure 2). The subsequent sections of this review paper will delve deeper into the specific applications and advancements in clinical informatics for BPD, including definitions, phenotyping, prediction models, and interventions, contributing to the ongoing goal of mitigating the impact of BPD on premature infants.

## 6 BPD redefined: A focus on phenotypic characteristics

BPD is a heterogeneous condition in terms of its pathophysiology, clinical presentation, and treatment response. Hence, there is a compelling rationale for categorizing BPD patients into distinct subgroups or phenotypes based on their unique clinical and molecular profiles. Phenotypes are artificial constructs consisting of observable characteristics or traits of disease (53). Traditionally, BPD severity has been classified based on the requirement for oxygen therapy or positive pressure ventilation at 28 days and/or 36 weeks postmenstrual age (3, 54). However, these approaches fail to capture the heterogeneity of the disease and its underlying mechanisms.

Clinical phenotyping offers an alternative strategy for optimizing therapeutic interventions and refining risk stratification, mirroring the successful subcategorization of conditions like asthma based on clinical presentation. This approach enables more precise guidance for tailored therapies (55). The intricate interplay of prenatal risk factors, including but not limited to pregnancy-induced maternal hypertension, intrauterine growth restriction, genetic predisposition, chorioamnionitis, and other causes of fetal systemic inflammation, creates a dynamic landscape that influences the future clinical phenotype of a neonate (56, 57). These factors, combined with postnatal influences such as prematurity, birth weight, oxidative stress, mechanical ventilation, sepsis, the presence of a clinically significant patent ductus arteriosus, and respiratory microbial dysbiosis, contribute not only to the development of BPD but also shape the challenges clinicians must address. This interplay informs the need for tailored interventions to meet the unique needs of each neonate (58).

An approach to classifying BPD based on phenotypes holds considerable clinical implications, such as clinical trial recruitment, retrospective cohort analysis, outcome studies, and cost analyses (5, 6). Recent studies have applied clustering techniques to describe BPD phenotypes based on various clinical and molecular features. In a study of 76 neonates with severe BPD, Wu et al. stratified phenotypic presentations according to 1) moderate-severe parenchymal lung disease, 2) pulmonary hypertension, and 3) large airway disease (59). The authors defined parenchymal lung disease by scoring severity via chest tomography with angiography. Specifically, the criteria assessed the existence and intensity of ten distinct lung radiographic attributes, employing a scale that spans from 0 to 2. The attributes include hyper-expansion, mosaic attenuation pattern, intercostal bulging, air cysts, bullae, blebs, cyst size, triangular subpleural opacities, distortion and thickening of bronchovascular bundle, consolidation, and a subjective assessment of overall lung disease severity. Pulmonary hypertension was defined by having one or more of the following: a systolic pulmonary artery pressure ≥40 mm Hg, a bidirectional or right-to-left shunt through a patent ductus arteriosus, or a flattened or bowing interventricular septum at the end of systole. Large airway disease included tracheomalacia and/or bronchomalacia documented on bronchoscopy and/or tracheoscopy by a pediatric otolaryngologist or pulmonologist. Outcomes were then described according to the three prespecified phenotypes. Neonates with pulmonary hypertension or large airway disease phenotypes had higher odds of mortality, tracheostomy, or pulmonary vasodilator use at discharge when compared to those classified with parenchymal lung disease (OR 5.4 vs. 5.1 vs. 0.59, respectively).

bpd phenotyping plays a crucial role in not only enhancing the precision of BPD definition but also in improving clinical care and guiding future intervention studies. By employing unsupervised machine learning algorithms, we can achieve a finer stratification of BPD severity, allowing for more tailored and effective therapeutic approaches. Additionally, it is imperative that future models consider the dynamic shifts in clinical phenotypes that occur as interventions are administered or discontinued during the course of hospitalization. This adaptive approach ensures that the therapeutic strategies remain aligned with the evolving needs of the neonate.

## 7 BPD predictive models and support tools

### 7.1 Predicting extubation success in non-BPD patients

Minimizing the number of mechanical ventilation days and the need for multiple courses of mechanical ventilation is one of the major initiatives aimed at preventing the development of BPD and/or decreasing its severity (60). However, early extubation to prevent BPD must be balanced with the risks of failed extubation and increased morbidity and mortality in extremely preterm infants (61, 62). This is especially challenging for the highest-risk group of 22–24-week gestational-age infants. In the Swedish national quality registry, which has over 50% survival for 22-week gestational age infants, the median duration of mechanical ventilation was 54 days for 22-week GA infants, 32 days for 23-week infants, and 22 days for 24-week infants (63). While several prediction models using logistic regression or ML methods have been developed to aid clinicians in determining the appropriate timing for extubation in extremely preterm infants, the models do not include many patients in the high-risk category of 22–24 weeks GA and none have been routinely adopted into clinical practice (64–68). Moreover, it is worth noting that the extubation prediction models available have been developed for patients who are at risk of developing BPD and not for patients who have already been diagnosed with the condition (69). Therefore, the development of extubation prediction models for neonates with BPD could prove to be even more valuable.

### 7.2 Predicting extubation success in BPD patients

The timeline for diagnosing BPD and its impact on the development of extubation prediction models is indeed a crucial aspect to consider. Currently, the diagnosis of BPD is typically made based on clinical criteria, often at 28 days or 36 weeks postmenstrual age. In the context of developing extubation prediction models, this timeline poses challenges. Timing of extubation models should take into consideration how the diagnosis of BPD impacts their utility. To address this gap, it is essential for these models to take into account the dynamic shifts in clinical phenotypes that occur during the course of hospitalization. Several strategies for achieving this include:

#### 7.2.1 Data collection

Comprehensive data collection, including clinical, physiological, and treatment-related variables, from neonates with diagnosed BPD is essential. This data should encompass the entire hospitalization period and should be routinely updated to reflect changes in the patient's condition.

#### 7.2.2 Machine learning algorithms

Machine learning algorithms, particularly those with adaptability and the ability to handle dynamic data, should be employed. These algorithms can be trained on the collected data to make real-time predictions about the optimal timing for extubation for individual patients.

#### 7.2.3 Continuous monitoring

An important aspect of these models is continuous monitoring of the patient's clinical status. As interventions are administered or discontinued, the models should adapt and refine their predictions based on their clinical changes.

#### 7.2.4 Collaboration

Collaboration among healthcare institutions is crucial. By aggregating data from a larger cohort of neonates with BPD, we can enhance the generalizability and accuracy of these models.

Additionally, it is worth noting that as of now, no randomized control trials have investigated extubation practices for neonates with BPD. Consensus guidelines recommend ventilator practices that include a slow rate, high tidal volume (7–12 ml/kg), and prolonged inspiratory times (>0.6 s) to account for the varying levels of airway resistance and altered distal lung compliance, which leads to diverse and different time constants (7). Furthermore, extubation should be considered once infants are on a stable low oxygen requirement, which indicates adequate lung growth and healing rather than relying on data such as carbon dioxide or end-tidal carbon dioxide (7).

Some centers have a standardized approach for deciding when to attempt extubation or consider tracheostomy for patients with severe BPD. In the Bronchopulmonary Dysplasia collaborative, only five of the 15 institutions (33%) have a ventilator weaning protocol or extubation protocol (70). Artificial intelligence-based algorithms, such as ML models, may assist in suggesting ventilator weaning strategies, predicting extubation success, or the probability of long-term mechanical ventilation. Advanced deep learning ML algorithms may be necessary to process all the complex data required for prediction, such as dynamic ventilator settings, pulse oximeter saturation and stability, blood gas results, growth velocity, chest x-ray image analysis, and other relevant factors.

### 7.3 Clinical decision support tools for BPD management

There has been a recent emphasis on developing clinical decision support (CDS) tools to predict and prevent BPD. While many prediction models have been developed, no single tool has been universally adopted. A recent systematic review and meta-analysis identified 64 studies that developed or validated 53 BPD prediction models, which incorporated perinatal factors such as gestational age and birth weight, along with specific clinical features evaluated at different timepoints (5). However, the determination of significant predictors and BPD definitions varies across regions and patient cohorts, underscoring the importance of developing and validating prediction tools in neonatal networks worldwide (71–73).

There are several advantages to the shift toward using informatics to build models to predict BPD and its outcomes. In the 2021 study by Jasseem-Bobowicz et al, a prediction model was created using clinical factors including gestational age, number of RBC transfusions, number of surfactant administrations, and patent ductus arteriosus data. These factors were determined by machine learning tools including regression coefficients to establish a categorical risk scoring system that was then used to create risk categories for this model (74). The advantage of this study was its performance (AUC 0.932) and its simplicity and ease of use in clinical practice. However, drawbacks include a single-center was used to build the model, limiting generalizability. In a more recent 2023 study by Gao et al, multivariate logistic regression analysis using a stepwise fashion was performed for risk factor selection, and the least absolute shrinkage and selection operator (LASSO) was carried out for factor selection. A nomogram model was developed using a R Package “rms” to predict BPD outcomes. This model also did well with an AUC of 0.910 in the training and 0.9051 in the validation cohort. The strengths of this study were the comparison of two predictor selection methods and external validation of the risk factors, while a limitation included a small sample size from one institution (75). Another recent 2023 study by Ou et al. also used the LASSO method by 5-fold cross-validation to select the most useful predictive proteins for BPD prediction. The protein model yielded an AUC of 0.96 in the test cohort (76). The advantages of this approach included a computational and molecular integration to predict BPD. Overall, there have been more than 100 prediction models that have been developed to predict BPD, as summarized in a systemic review by Romjin et al. (77).

While numerous prediction models for BPD have been developed, their full potential remains underutilized due to several key challenges. One of the primary limitations is the lack of external validation (6). For a CDS tool to be truly effective, it must not only provide accurate predictions but also demonstrate its generalizability and reliability in real-world clinical settings. External validation, often referred to as replication, plays a pivotal role in this process, as it involves testing prediction models with data generated independently from the dataset used for model creation. One promising example in the field is the National Institutes of Childhood and Health Development (NICHD) Neonatal Research Network BPD Outcome Estimator, which employs a series of multinomial logistic regressions incorporating six critical risk factors to predict the severity of BPD or mortality (78–80). The model was internally and externally validated; however, it is still associated with racial and regional bias. To enhance its clinical translation and overcome these biases, efforts have been made to create the online “BPD Estimator” and update it in 2022 with a more recent dataset and refined BPD definition (80).

To address the current limitations and promote wider adoption of such prediction tools, a multifaceted approach is needed. This should include:

#### 7.3.1 Ongoing external validation

To ensure the reliability and generalizability of prediction models for BPD, it is imperative to conduct ongoing external validation. This process involves testing the models using diverse patient populations and across various healthcare settings, including different regions and institutions. By doing so, we can assess how well these models perform in real-world scenarios, which may vary in terms of patient demographics, clinical practices, and environmental factors. This continuous validation process not only enhances the models’ accuracy but also builds trust among healthcare providers, making them more likely to integrate these tools into their clinical decision-making processes.

#### 7.3.2 Continuous evaluation of model performance

The development of prediction models should not be a one-time effort. Instead, it should be an iterative process that involves continuous evaluation and refinement. Regularly assessing model performance is crucial to identifying changes in accuracy over time, potential biases that may emerge, or changes in the patient population that the model serves. This evaluation should be data-driven and should lead to updates and improvements in the models as new data become available. In essence, it is about ensuring that the prediction tools remain relevant and effective.

#### 7.3.3 Mitigating biases and disparities

Recognizing and addressing biases within prediction models is important. Models should be carefully scrutinized to identify any biases related to race, ethnicity, socioeconomic status, or geographic location. These biases can lead to disparities in healthcare outcomes. Mitigating biases involves refining the algorithms, diversifying the training datasets, and incorporating fairness and equity considerations into model development. By actively working to reduce biases, we can ensure that these prediction tools provide equitable care to all neonates, irrespective of their background or location.

#### 7.3.4 Promoting awareness and education

One significant barrier to the adoption of prediction tools is a lack of awareness and understanding among healthcare providers. Efforts should be made to educate neonatal care teams about the availability, benefits, and utility of these tools. This can involve publishing in reputable journals and presenting data in conferences or workshops, with the eventual goal of integrating into EHRs. When healthcare providers are well-informed about the potential of these tools to enhance patient care, they are more likely to incorporate them into their clinical workflows, ultimately benefiting neonates at risk of BPD.

While a single intervention to prevent BPD has been elusive, there are likely adaptable strategies across fetal, neonatal, infant, and childhood stages that can have a tangible summative effect (81). The strategies could target crucial developmental windows that our current research/QI methodologies might overlook. Leveraging artificial intelligence/machine learning/deep learning techniques can enhance our understanding by discerning patterns from pertinent and complex clinical and biological data and provide CDS tools to clinicians over a care continuum.

An illustrative example of clinical informatics’ impact can be observed in the field of oncology. In cancer care, clinical informatics has enabled the integration of genetic and clinical data tools to facilitate the identification of specific cancer subtypes and the tailoring of treatments to individual patients. For instance, the Molecular Analysis for Therapy Choice (MATCH) trial, conducted by the National Cancer Institute (NCI), employs informatics-driven genomic profiling to match cancer patients with targeted therapies, resulting in more effective treatments and improved patient outcomes (82).

In the field of critical care medicine, clinical informatics has revolutionized patient monitoring and early condition detection. For instance, the implementation of predictive analytics and clinical decision support systems has significantly improved patient management in ICUs. The eICU Collaborative Research Database, a notable example, collects data from thousands of ICU patients across the United States and employs informatics-driven algorithms to predict adverse events, allowing healthcare teams to intervene proactively and reduce mortality rates (83).

Translating this success to neonatology, clinical informatics holds the potential to develop predictive models and clinical decision support tools that assist neonatal care teams in optimizing care strategies for infants at risk of BPD. By analyzing complex data from NICUs, these tools can provide early warnings of deteriorating conditions, helping clinicians take timely actions to prevent or mitigate the severity of BPD.

At present, available CDS tools for BPD still have several limitations. Often, these models were created with data from a single center, have a small sample size, lack good quality external validation, and may only apply to specific high-risk infants such as those on ventilators, limiting their generalizability. Moreover, over half of the published models use data from infants born over a decade ago, which may not reflect present-day clinical practice (6). Additionally, existing models do not account for the infant's clinical trajectory over time, which could provide a dynamic approach for better personalized preventive treatment and targeted trial recruitment. Nonetheless, a recent machine learning (ML)-based prediction model demonstrated the predictive power of postnatal respiratory support for 14 consecutive days (84). While this model was developed at a single center without validation, it demonstrates how prediction models and tools are evolving to better meet clinical needs.

To address the limitations of current CDS tools for BPD, there are several potential solutions that can be considered. First, efforts should be made to collect and integrate data from multiple centers to create more robust and generalizable models. Collaborative initiatives can help gather a larger and more diverse dataset, ensuring that the models are applicable across different clinical settings. Furthermore, it is crucial to validate these models externally to confirm their accuracy and reliability in various populations. Additionally, updating the datasets with recent clinical data is essential to reflect the current clinical practice, as BPD management and patient characteristics evolve over time. To provide a more dynamic approach, future models can incorporate the infant’s clinical trajectory over time, enabling personalized preventive treatment strategies and more targeted recruitment for clinical trials. While the recent ML-based prediction model for postnatal respiratory support is a step in the right direction, future research should focus on refining and validating such models to ensure their clinical utility and effectiveness across multiple healthcare systems. This collaborative and evolving approach to CDS tools can significantly enhance their effectiveness in managing BPD.

## 8 Use for prevention of BPD or decreasing its severity

Clinical informatics has the potential to make significant strides in the prevention and reduction of the severity of BPD among neonates. One of the key applications is early risk prediction. Informatics tools can analyze a multitude of data points, including gestational age, birth weight, respiratory parameters, and maternal health records, to identify neonates at high risk of developing BPD. By flagging these high-risk cases, healthcare teams can implement preventive measures promptly, such as optimizing respiratory support strategies or initiating lung-protective ventilation strategies. In addition to early risk prediction, clinical informatics can also play a crucial role in improving communication and coordination among healthcare providers involved in the care of neonates with BPD. Informatics tools can facilitate seamless sharing of patient data, treatment plans, and progress updates among neonatologists, pulmonologists, nurses, and other healthcare professionals. This enhanced collaboration can lead to more effective and timely interventions, ultimately reducing the severity of BPD (4).

Clinical informatics can offer a valuable tool in the form of automated weekly reminders and graphical representation of ventilator parameters to assess respiratory progress in neonates with BPD. These reminders can prompt healthcare providers to systematically evaluate the infant’s respiratory status on a regular basis, ensuring that no critical changes or deteriorations go unnoticed. Furthermore, informatics systems can generate graphical displays of ventilator parameters over the past few weeks, allowing clinicians to visualize trends and deviations in respiratory support. Such visualizations can highlight improvements or declines in a neonate's lung function, enabling healthcare teams to make informed decisions regarding adjustments to ventilation strategies or other therapeutic interventions. This proactive approach not only supports timely interventions but also aids in fine-tuning treatment plans to optimize respiratory care and minimize the severity of BPD.

Informatics systems enable continuous monitoring and data analysis of neonatal patients in real-time. By tracking physiological parameters, like oxygen saturation levels and respiratory rates, clinical informatics can detect subtle changes that may indicate evolving BPD (85). When these early warning signs are identified, healthcare providers can adjust treatment plans and interventions, potentially preventing or mitigating the progression of the disease (86). Another critical aspect is the personalization of care plans. Clinical informatics can assist in tailoring therapeutic approaches based on individual patient data. For instance, informatics tools can analyze a neonate's response to specific treatments and medications, enabling the adjustment of therapy to maximize effectiveness while minimizing potential side effects. This personalized approach can optimize outcomes and decrease the overall severity of BPD (47).

## 9 Use in those with already established BPD

Clinical informatics can also contribute to the care of neonates with established BPD by streamlining medication management, integrating clinical data, enabling remote consultations, supporting quality assurance efforts, and engaging patients and their families.

### 9.1 Enhanced medication management

Clinical informatics provides a platform for the management of medications in neonates with established BPD. It assists clinicians in maintaining accurate and up-to-date medication records, tracking dosages, and monitoring adverse effects. This ensures that infants receive their prescribed medications consistently and safely. Moreover, informatics systems can generate alerts for potential drug interactions or dosage adjustments, helping clinicians make informed decisions regarding pharmacological interventions (87).

### 9.2 Efficient data integration

Managing the care of neonates with established BPD often involves a multitude of clinical data sources, including laboratory results, imaging reports, and patient histories. Clinical informatics excels at integrating these data sets into a unified and accessible format. It allows clinicians to access a comprehensive view of each patient's health status, and prior response to medications/treatments, streamlining decision-making and reducing the risk of oversight (88).

### 9.3 Remote consultation and telehealth

In situations where neonates with established BPD require specialized care or consultations from experts, clinical informatics facilitates remote consultations and telehealth services. Clinicians can securely share patient data and images with specialists, enabling timely expert opinions and guidance without the need for physical presence. This extends access to expertise and can be particularly valuable in managing complex cases (89).

### 9.4 Quality assurance and benchmarking

Clinical informatics offers tools for quality assurance and benchmarking in the care of neonates with BPD. Clinicians can compare their treatment outcomes and practices with national or international standards and benchmarks. This data-driven approach allows for continuous improvement in care protocols, ensuring that the latest evidence-based practices are adopted to enhance patient outcomes (90).

### 9.5 Patient and family engagement

Informatics systems also have a role in engaging patients and their families in the care process. Neonates with established BPD often require extended hospital stays, and informatics can provide a means for families to stay informed about their infant's progress. It offers patient portals and educational resources to empower families with information and facilitate communication with clinicians, promoting a collaborative approach to care (91).

## 10 BPD registries with full integration with EHR

Current data collection and storage methods supporting BPD research are varied. Some databases aim to collect general demographic, clinical, and outcomes data on very low-birthweight infants, while others are dedicated to sponsored multi-site trials investigating BPD as a primary or secondary outcome. Additionally, some databases focus on more granular data derived from a single-site center. Large databases targeting VLBW infants exist both in the US and internationally, and most data is entered manually by principal investigators or trained data abstractors at participating sites (92–96). However, the United Kingdom's National Neonatal Database stands out as an exception due to its use of a common EHR shared among participating sites, allowing data to flow automatically into the database on a quarterly basis. Quality assessment of the extracted data is then published for participants to review (95). A preliminary search conducted on PubMed on January 8, 2024, using the keywords “National Neonatal Research Database (NNRD)” returned 26 publications, primarily centered around neonatal nutrition. Surprisingly, none of the studies identified in this initial search were specifically focused on BPD. We believe future research questions will delve into the utilization of the NNRD to investigate BPD's epidemiology, risk factors, outcomes, and potential interventions.

Despite the existence of these databases, challenges remain in data collection, including the labor-intensive nature of manual data entry, incomplete data, evolving definitions of disease, inability to capture longitudinal data, inherent data bias, data governance, and privacy concerns. These challenges highlight the need for continued work on improving data collection and storage methods to support BPD research (refer to Table 1).

**Table 1:** Table of select existing national registries of neonates with or at risk for BPD.

Database name	What works	What does not work	What needs to happen to enhance utility
Generic Database of VLBW infants	Comprehensive data collection Demographics, medical outcomes data Wide participant base (NICHD Neonatal Network Long-term data collection (1987–2030)	Limited integration with EHRs Concerns about prediction accuracy	Improved EHR integration Enhanced prediction accuracy Further data sharing and collaboration
NICHD DASH (Data and Specimen Hub)	Data sharing platform for completed studies Availability of biospecimens Control and experimental arms in some studies	Sample size limitations Convenience sampling	Expansion of studies and sample sizes Inclusion of more diverse study designs Greater diversity in participant recruitment
Vermont Oxford Network (VON)	Data collection from NICUs worldwide Benchmarking and comparative analysis Identifying variations in care and outcomes	Limited granularity Data definitions are strict Challenges in standardization of terms	Enhanced data granularity and specificity
International Network for Evaluation of Outcomes (iNEO) of Neonates	International collaboration in research Data-informed practice changes in NICUs Uniform definition database	Limited to specific weight and gestational age Mapping to ICD-10 and SNOMED-CT	Expansion of eligible neonates Continued impact assessment in participating networks Further data linkage and mapping to enhance interoperability
Canadian Neonatal Network	Insights into neonatal care practices in Canada Benchmarking reports for NICUs	Site-specific eligibility criteria	Broadening site eligibility and inclusivity Uniform definition across the database
United Kingdom National Neonatal Research Database	Extracts data from electronic patient records High completeness of patient characteristics Data mapped to ICD-10 codes	Data extraction is not real-time Limited geographical coverage Quarterly data extraction	Real-time data extraction for more current insights Expanded coverage to other regions Automated data extraction for efficiency
Swedish Neonatal Quality Register (SNQ)	Extensive data collection in Swedish neonatal units Covers a wide range of variables Includes key performance indicators	Data completeness variation Data collection for several years	Consistent and complete data collection
Korean Neonatal Network	Prospective web-based registry for VLBW neonates Unique systems for data display and monitoring	Data quality surveillance limited to site visits	Enhanced real-time data quality surveillance Continued emphasis on data quality and completeness
Chinese Neonatal Network	Uniform definition database Mapping terms to ICD-10 and SNOMED-CT Comprehensive data collection	Manual data extraction and data checks Sample-specific eligibility criteria	Streamlined data entry and automated checks Feedback loop for site-specific data quality improvement Inclusion of data elements

VLBW, very low birth weight; NICU, neonatal intensive care unit.

Moreover, without consensus on a minimum standard dataset, comparing and combining data across databases becomes challenging. In addition, the lack of standardized definitions and terminologies hinders cross-site analysis and increases the risk of errors in data interpretation. To address this, initiatives such as the Neonatal Research Network (NRN) have developed standardized definitions for outcomes and clinical variables, which have been adopted by many large databases. Despite these initiatives, there is still a need for more widespread adoption of standardized definitions and terminologies to improve interoperability and facilitate collaboration across different databases and research groups. The use of common data models, such as the OMOP CDM, may help in achieving this goal by providing a standardized structure for data collection and storage that can be shared across different institutions and studies.

Improving the collection and storage of BPD data is crucial for advancing our knowledge of the disease and enhancing patient outcomes. To achieve this goal, it is necessary to leverage automated data extraction and mapping of a minimum dataset to standard definitions and terms. The lack of BPD-specific data granularity in current VLBW databases limits their research potential in answering questions about candidate treatments or management approaches. Standardization of data elements has been recognized by some registries, but many remain unmapped to accepted standard dictionaries such as International Statistical Classification of Diseases and Related Health Problems-10 (ICD-10) or Systematized Nomenclature of Medicine-Clinical Terms (SNOMED-CT), which limits collaboration and interoperability (96, 97). The UK model demonstrates the potential for automated data extraction with high fidelity and provides a roadmap for streamlining epidemiologic data collection (98, 99). By better capturing demographic, clinical, and outcome data, we can perform retrospective investigations and explore disease incidence and risk factors. Ultimately, this can inform the development of improved management approaches and more effective treatments for patients with BPD.

## 11 Multi-Center collaborations in BPD research

Due to center-specific variations in patient care and outcomes, multi-center collaborations are necessary to advance our understanding of the disease, identify best practices, and improve patient outcomes (1, 70, 100). Multi-center collaborations offer numerous benefits in BPD research, including a better understanding of disease mechanisms, lung repair, and regeneration. Through these collaborations, disease phenotypes can be better classified and novel therapeutic targets can be better explored. Multi-center collaborations can identify variations in neonatal outcomes between centers, leading to national and international benchmarking, quality improvement activities, and implementation of “best practice” strategies (1, 7, 100).

Multi-center collaborations, exemplified by initiatives like the BPD Collaborative encompassing 27 centers across the USA and one in Sweden, have undeniably propelled BPD research forward and have improved patient care outcomes (7, 58, 100, 101). Within these collaborations, clinical informatics has emerged as a critical component, serving to enhance the efficiency and effectiveness of research endeavors. Clinical informatics systems provide a secure and standardized platform for the exchange of patient data and research findings among participating centers. This data sharing facilitates the aggregation of extensive datasets from diverse clinical settings, empowering researchers to conduct comprehensive analyses spanning multiple institutions. By harnessing the power of data integration, clinical informatics enables the identification of trends and insights that might otherwise remain elusive when working solely with isolated datasets. Furthermore, informatics tools play a central role in harmonizing clinical practices and standardizing data collection protocols across collaborating institutions, ensuring consistent data collection and adhering to predefined standards (58). This standardization minimizes variations in data quality and enhances the reliability of research outcomes. In the context of the BPD Collaborative, clinical informatics has been instrumental in facilitating the implementation of essential strategies, such as optimizing ventilator management and nutritional practices. By tracking patient responses and outcomes using informatics tools, the collaborative has identified best practices and areas for improvement, contributing to the enhanced quality of care and improved patient outcomes (100, 101).

In the study by Guaman et al, clinical informatics played a key role in understanding neonates with severe BPD. For instance, clinical informatics facilitated the uniform collection of clinical data across the eight NICUs. A standardized clinical data form was designed and implemented, ensuring that essential patient information, including gestational age, respiratory support requirements, and medication usage, was consistently recorded for all inpatients born at <32 weeks. The study defined severe BPD based on specific criteria, including the need for ≥30% supplemental oxygen and/or positive pressure ventilation at 36 weeks postmenstrual age. Clinical informatics played a key role in applying these criteria uniformly across participating NICUs. Clinical informatics also facilitated the analysis of management practices across the participating NICUs. The study examined differences in the use of interventions such as mechanical ventilation, diuretics, inhaled corticosteroids, and inhaled *β*-agonists. By collecting and analyzing these data using informatics tools, the researchers identified variations in the management of infants with severe BPD among different centers (102).

In addressing the significant challenges that persist in multi-center collaborations for BPD research, it is essential to recognize the multifaceted nature of this condition. The complex and multifactorial etiology of BPD, encompassing factors such as prematurity, inflammation, and genetic predisposition, contributes to the intricate clinical landscape (58, 100). Furthermore, BPD is not a one-size-fits-all condition. It presents a wide spectrum of patient characteristics, including variations in gestational age, birth weight, and comorbidities, necessitating diverse and tailored management approaches that can evolve over the course of a patient's hospitalization and even throughout their lifetime (103).

The challenges extend to data collection and management practices. Current data collection methods are labor-intensive and often fraught with incompleteness, preventing a comprehensive and real-world representation of the clinical phenotype of BPD (1, 79). Moreover, the evolving definitions of disease pose an ongoing challenge. The field continuously refines diagnostic criteria and treatment strategies, making it critical to capture longitudinal data to track changes in patient care and outcomes over time. However, this longitudinal perspective remains elusive due to existing data limitations.

Data governance and privacy concerns also loom large, impeding the seamless sharing of critical information among institutions. Protecting patient privacy while facilitating data sharing demands sophisticated solutions and adherence to stringent regulations (1, 79). Additionally, BPD lacks a universally accepted “single” therapy, further complicating research efforts. Tailoring treatments to individual patients requires nuanced real-world data to inform decision-making and optimize care.

Efforts such as the OHDSI (Observational Health Data Sciences and Informatics) OMOP CDM (Common Data Model) present a promising avenue to overcome the substantial challenges in multi-center collaborations for bronchopulmonary dysplasia (BPD) research (104). The OHDSI initiative is at the forefront of harnessing clinical informatics to standardize and streamline data collection and analysis. The OMOP CDM, an integral component of OHDSI, serves as a standardized clinical data framework that allows the extraction, ingestion, and collation of pertinent variables from diverse sources, enabling the creation of disease-specific observational research registries like a BPD registry (105, 106).

Central to the success of these initiatives are widely recognized clinical terminologies, such as SNOMED CT (Systematized Nomenclature of Medicine Clinical Terms) and LOINC (Logical Observation Identifiers Names and Codes) (107, 108). SNOMED CT provides a comprehensive and standardized clinical vocabulary that enhances interoperability and consistency in healthcare data, while LOINC facilitates the uniform identification of medical laboratory observations, ensuring data accuracy and comparability across institutions.

These standardized clinical data frameworks empower researchers to securely store data, conduct rigorous analyses, and efficiently share information among participating sites, thereby reducing the barriers to collaboration and facilitating data-driven research efforts. The concept of a “living BPD registry,” driven by OHDSI OMOP CDM and bolstered by SNOMED CT and LOINC, holds the potential to revolutionize BPD research and collaborative endeavors. It offers the opportunity for reproducible research in phenotype definitions and comparative analyses, leveraging a wealth of standardized real-world data across institutions. Ultimately, these initiatives not only lower the activation energy required for collaboration but also hold the promise of driving meaningful advancements in BPD care and research outcomes.

## 12 Challenges and future directions in BPD research and clinical informatics

BPD research and the integration of clinical informatics have undoubtedly made significant progress, yet several formidable challenges persist (106). The current definition of BPD, based on respiratory support at 36 weeks postmenstrual age, represents a crucial step forward in standardizing diagnostic criteria and understanding long-term patient-centered outcomes (79). However, it remains an oversimplified representation of the intricate clinical reality and heterogeneity that exists within the BPD spectrum.

To address these challenges effectively, it is imperative to delve into the nuances that distinguish children with varying degrees of BPD severity. A more granular understanding can guide the development of interventions tailored to specific subgroups of patients while minimizing potential harm. This nuanced approach acknowledges the multifaceted nature of BPD and recognizes that each neonate’s clinical phenotype is unique.

Despite the existence of risk stratification tools for BPD, their full potential has not been realized due to limited integration with EHRs and concerns regarding prediction accuracy. This stands in stark contrast to the robust adoption of other neonatal tools, such as those used for predicting early-onset sepsis or guiding phototherapy recommendations (52, 78). To comprehensively address the complexities of defining, preventing, and treating BPD, CDS tools must not only seamlessly integrate with EHRs but also offer comprehensive treatment guidance. Achieving this requires a strategic approach focused on tailored care rather than advocating for a one-size-fits-all reduction in care.

Our proposed clinical informatics approach aims to empower clinicians with timely, individualized information for optimized decision-making. By harnessing data-driven insights and predictive models, we provide care providers with the tools to make informed decisions that align with each neonate's specific risk profile. This approach takes into account both clinical severity and individual risk factors. While some moderate-risk neonates may indeed benefit from escalated care, applying the same approach universally could lead to overutilization of resources and unnecessary interventions. Clinical informatics enables the identification of moderate-risk neonates most likely to benefit from escalated care, thereby maximizing the utility of interventions while minimizing potential harm or resource inefficiency.

Collaborative efforts involving multiple centers are imperative for advancing our understanding of BPD. Such collaborations bring together larger sample sizes, diverse patient populations, and a broader range of expertise. Nevertheless, challenges related to data collection, storage, and the lack of standardization of terms and definitions persist. To facilitate seamless collaboration and interoperability, the standardization of data elements and mapping to recognized clinical terminologies such as ICD-10 and SNOMED-CT is essential. Additionally, the establishment of a “living BPD registry” is crucial, capable of efficiently collecting and storing data while incorporating more granular information such as clinical biomarkers, echocardiography/radiographic data, and high-resolution vital signs and ventilator data. This approach enables more accurate clinical phenotyping, thereby advancing our understanding of BPD and facilitating tailored care.

## 13 Conclusion

In conclusion, BPD is a complex disease that affects premature infants and is responsible for significant morbidity and healthcare costs. While clinical informatics holds promise in improving BPD prevention, management, and outcomes through the use of clinical decision support tools, we are still in the very early stages of using these applications effectively. Research efforts utilizing multi-center collaborations and state-of-the-art analysis techniques may help identify clinical phenotypes of BPD. However, many challenges remain in BPD research and clinical informatics, including the need for standardized data collection and definitions, full integration with EMR, the development of precise and personalized treatments for different BPD subtypes, and the logistics and governance issues with data storage, utilization, and collaboration. Overall, while there is promise in the integration of clinical informatics into BPD management and research, much work remains to be done to fully realize its potential in improving outcomes for these vulnerable patients.
